# Association between TyG index trajectory and new-onset lean NAFLD: a longitudinal study

**DOI:** 10.3389/fendo.2024.1321922

**Published:** 2024-02-27

**Authors:** Haoshuang Liu, Jingfeng Chen, Qian Qin, Su Yan, Youxiang Wang, Jiaoyan Li, Suying Ding

**Affiliations:** ^1^ Health Management Center, the First Affiliated Hospital of Zhengzhou University, Zhengzhou, China; ^2^ College of Public Health, Zhengzhou University, Zhengzhou, China

**Keywords:** triglyceride-glucose index, latent class growth model, lean nonalcoholic fatty liver disease, health management, trajectory

## Abstract

**Objective:**

The purpose of this manuscript is to identify longitudinal trajectories of changes in triglyceride glucose (TyG) index and investigate the association of TyG index trajectories with risk of lean nonalcoholic fatty liver disease (NAFLD).

**Methods:**

Using data from 1,109 participants in the Health Management Cohort longitudinal study, we used Latent Class Growth Modeling (LCGM) to develop TyG index trajectories. Using a Cox proportional hazard model, the relationship between TyG index trajectories and incident lean NAFLD was analyzed. Restricted cubic splines (RCS) were used to visually display the dose-response association between TyG index and lean NAFLD. We also deployed machine learning (ML) via Light Gradient Boosting Machine (LightGBM) to predict lean NAFLD, validated by receiver operating characteristic curves (ROCs). The LightGBM model was used to create an online tool for medical use. In addition, NAFLD was assessed by abdominal ultrasound after excluding other liver fat causes.

**Results:**

The median age of the population was 46.6 years, and 440 (39.68%) of the participants were men. Three distinct TyG index trajectories were identified: “low stable” (TyG index ranged from 7.66 to 7.71, n=206, 18.5%), “moderate stable” (TyG index ranged from 8.11 to 8.15, n=542, 48.8%), and “high stable” (TyG index ranged from 8.61 to 8.67, n=363, 32.7%). Using a “low stable” trajectory as a reference, a “high stable” trajectory was associated with an increased risk of lean-NAFLD (*HR*: 2.668, *95% CI*: 1.098-6.484). After adjusting for baseline age, WC, SBP, BMI, and ALT, HR increased slightly in “moderate stable” and “high stable” trajectories to 1.767 (*95% CI*:0.730-4.275) and 2.668 (*95% CI*:1.098-6.484), respectively. RCS analysis showed a significant nonlinear dose-response relationship between TyG index and lean NAFLD risk (*χ^2 = ^* 11.5, *P*=0.003). The LightGBM model demonstrated high accuracy (Train AUC 0.870, Test AUC 0.766). An online tool based on our model was developed to assist clinicians in assessing lean NAFLD risk.

**Conclusion:**

The TyG index serves as a promising noninvasive marker for lean NAFLD, with significant implications for clinical practice and public health policy.

## Highlights

- NAFLD can occur in lean individuals and is often associated with low awareness in the general population.
- Patients with lean NAFLD have worse outcomes than the general NAFLD population.
- Development of newer marker targets for lean NAFLD patients for use in future trials.

## Introduction

Nonalcoholic fatty liver disease (NAFLD) is the most prevalent chronic liver disease worldwide and increases the risk of atherosclerosis, cardiovascular disease (CVD), liver cirrhosis, and liver cancer (1). Obesity is a key factor in NAFLD (2). Most mathematical prediction models for NAFLD/NASH are based on obesity and diabetes prevalence (3). 10–20% (4)of NAFLD patients who are neither overweight nor obese (BMI< 25 kg/m^2^ or< 23 kg/m^2^ in Asians) have “lean NAFLD”. Lean NAFLD is a primary source of cryptogenic liver disease (5), and lean people with NAFLD are more likely to have impaired glucose tolerance, hypertension, metabolic syndrome, and cardiovascular mortality (6). Therefore, early detection of patients at risk for lean NAFLD in a simple and effective manner is critical. However, the pathogenesis of lean NAFLD remains uncertain.

As the gold standard for diagnosing lean NAFLD, histopathological examination of liver biopsy has various limitations, such as invasiveness, poor acceptability, and a higher cost (7). Therefore, a non-invasive diagnostic approach for lean NAFLD is urgently needed. The combination of serum markers and other indicators is valuable for screening diseases due to their convenience, low cost and accurate diagnosis. Insulin resistance (IR) is involved in the pathogenesis and progression of lean NAFLD (8). Homeostatic model assessment for IR (HOMA-IR) is widely utilized in IR-related disorders and is the gold standard for diagnosing lean NAFLD (9). The triglyceride glucose (TyG) index has been described as a reliable and simple surrogate for measuring IR and as having substantial relevance in metabolic disorders such as NAFLD (10). However, most of these studies use a single TyG index measure to predict lean NAFLD risk, which omits a possible variability in TyG index trajectories over time.

Machine learning (ML), an emerging component of artificial intelligence, is gradually being implemented in the analysis of healthcare data. It serves as an influential tool for assisting the clinical decision-making process (11, 12). A statistical and comprehensive review of ML in medical diagnosis by Swanson et al. shows that ML technologies help medical professionals reduce diagnostic errors, improve health care delivery, and reduce treatment costs (13). Therefore, we conducted a lean NAFLD cohort study to capture longitudinal TyG index change trajectories from 2017 to 2019 and analyze the association between TyG index change trajectories and lean NAFLD risk through 2020.

We use an ML model to combine TyG index with common clinical features and predict the probability of patients with lean NAFLD. Subsequently, we developed a web-based calculator using the LightGBM model. This predictive tool can help physicians assess the risk of lean NAFLD patients and devise personalized medical strategies while optimizing the allocation of medical resources. In addition, we assessed the dose-response relationship between TyG index change trajectories and lean NAFLD risk. This study would suggest that TyG index trajectories over time may provide important clues to the development of lean NAFLD and a basis for health management and risk communication in primary care practices.

## Materials and methods

### Study population

**Figure 1:**
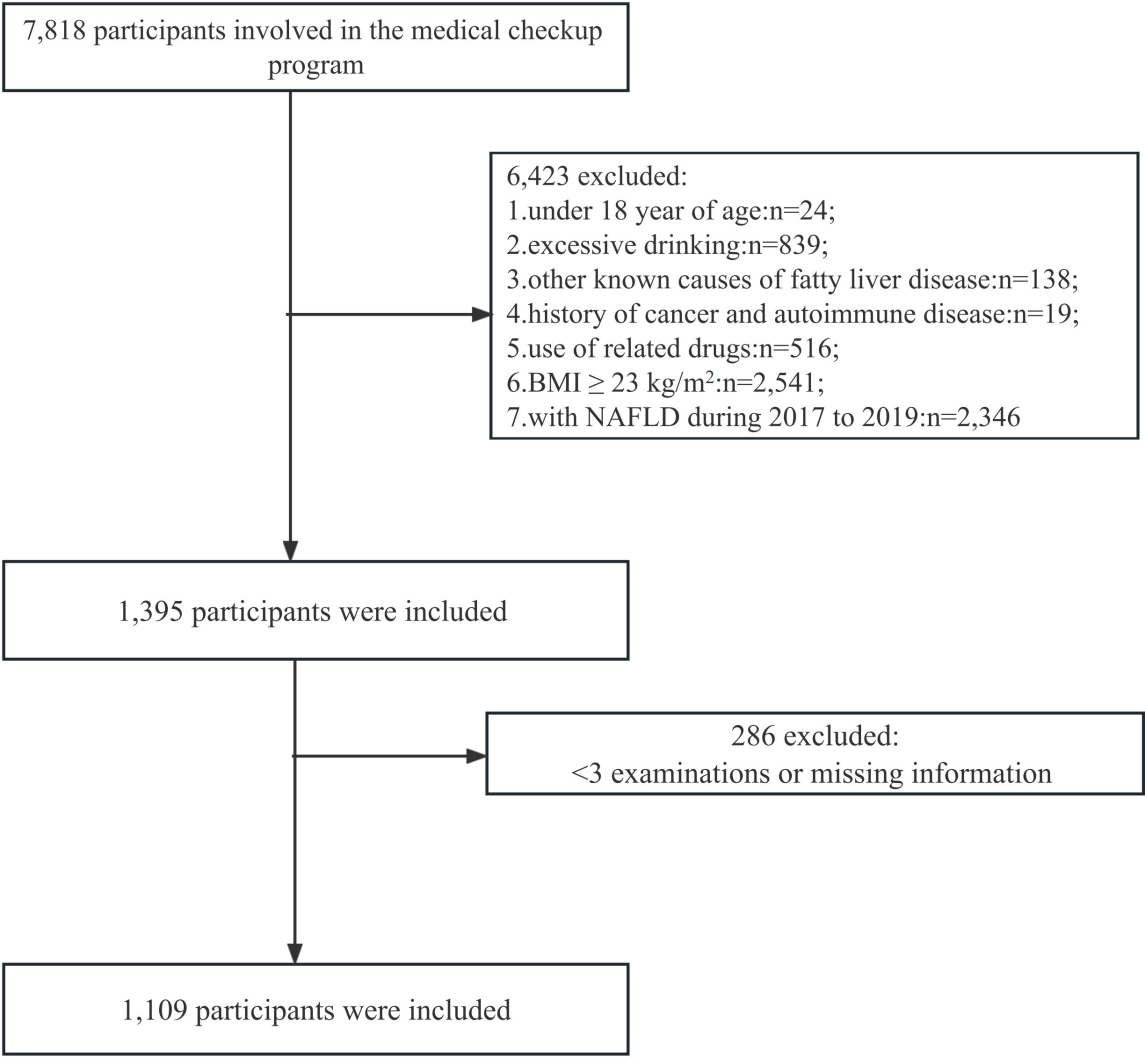
Flow chart of number of participants.

At the Health Management Center of the First Affiliated Hospital of Zhengzhou University, 7,818 individuals aged between 18 and 98 underwent health checks between 2017 and 2019. If a participant had liver ultrasound at baseline and at follow-up, they were recruited into the trial, and information about their medical history and medication use was collected. The following were the criteria for exclusion (1): aged< 18 years (n=24); (2) NAFLD diagnosis in 2017–2019 (n=2,346); (3) BMI ≥ 23 kg/m^2^ (n=2,541); (4) men and women who drink more than 10 and 20 grammes of alcohol daily, respectively (n=839); (5) history of fatty liver disease (n=138); (6) history of cancer or autoimmune disease (n=19); (7) usage of medications to reduce cholesterol, blood pressure (BP), blood sugar, or uric acid levels (n=516); and (8) less than three medical examinations or missing information in relevant variables (n=286). A total of 1,109 participants (441 males and 668 females) met the inclusion and exclusion criteria and were enrolled (**Figure 1**).

Following the Helsinki Declaration, this study was carried out. The study protocol was approved by the ethics committee of the First Affiliated Hospital of Zhengzhou University, Zhengzhou, China (approval No. 2018−KY−56). Written informed consent was obtained from all participants.

### Diagnostic criteria

We used two clinical ultrasound systems, ACUSON Oxana2 ultrasonic diagnostic device (Siemens Medical Solutions USA, CA 94043, USA) and 5-12 MHz probe (APLI0500TUS-A500, TOSHIBA, Tokyo, Japan), for direct post-beamform radio frequency (RF) data acquisition provided under research agreements. Three registered diagnostic medical sonographers (each with > 10 years of overall experience) were trained and had several months to 2 years of experience performing the research protocol. Each participant was scanned by one of the three selected sonographers based on scheduling availability and underwent two 15-20-minute exams on the same day. Each exam was performed using a different platform in random order. Between exams, participants took a 5-10-minute break and were repositioned on the gurney. An average of 50 patients are screened per day.

NAFLD was identified using Chinese criteria (14)-(1) enhanced echogenicity in the near field and attenuated echogenicity in the far field of the liver parenchyma; (2) hepatomegaly is characterized by relatively opaque intrahepatic ducts and rounded liver margins; (3) alcohol consumption of ≤ 140 g/week for men and ≤ 70 g/week for women; and (4) lack of other illnesses or disorders, such as viral hepatitis, total parenteral nutrition, hepatomegaly, and drug-related liver disease, which may lead to fatty liver disease. AGA clinical practice recommends the diagnosis of lean NAFLD in individuals with NAFLD and a BMI< 25 kg/m^2^ (non-Asian race) or< 23 kg/m^2^ (Asian race) (15). The BMI and TyG indices were calculated using the following formulas (16):


$$\text{BMI}=\text{body weight}\left(\text{kg}\right)/\text{body }{\text{height}}^{2}{\left(\text{m}\right)}^{2}$$

$$\begin{array}{c} \text{TyG }=\text{ Ln }\left[\text{fasting triglyceride }\left(\text{mg}/\text{dl}\right)\;\times \text{ fasting plasma glucose }\left(\text{mg}/\text{dl}\right)/2\right],(\text{TG}:1\text{mmol}/\text{L}\\ =88.5\text{mg}/\text{dL;FPG:}1\text{mmol}/\text{L }=18\text{mg}/\text{dL)} \end{array}$$

### Anthropology and laboratory investigations

During the physical examination, the patient’s height, weight, waist circumference (WC), systolic blood pressure (SBP), and diastolic blood pressure (DBP) were all measured. Height, weight, and WC were measured with participants dressed in light, thin clothing without shoes. BP was measured on the right arm of seated individuals using electronic sphygmomanometers.

Individuals were sampled following a 12-hour overnight fast. Fasting laboratory analysis included measurements of triglyceride (TG), total cholesterol (TC), low-density lipoprotein (LDL-C), high-density lipoprotein (HDL-C), fasting plasma glucose (FPG), glycated hemoglobin (HbA1c), aspartate aminotransferase (AST), alamine aminotransferase (ALT), γ-glutamyl transpeptidase (GGT), serum uric acid (SUA), hemoglobin (Hb) and white blood cell (WBC). The samples were analyzed using a chemical analyzer (cobas8000, Switzerland) based on enzymatic method at the hospital’s central laboratory. FIB-4 was calculated as age (year) x AST (U/L)/[platelet count (109/L) x √ALT (U/L)] (17). The Fibrotic NASH Index (FNI) can be calculated online (18).

### Questionnaire survey

Information on smoking status (current vs. never smokers) and drinking status (current drinkers vs. never drinkers vs. drinking amount) was obtained from self-reported data.

### Statistical analysis

First, descriptive analysis findings are reported as mean ± standard deviation for normally distributed continuous variables, medians (interquartile range) for non-normally distributed continuous variables, and number with percentage frequency for categorical variables. Comparisons of participants’ general characteristics at baseline were assessed using the Student’s t test for normally distributed continuous variables, Mann-Whitney U test for non-normally distributed continuous variables, and Chi-square test for categorical variables. Second, variables with a *P* value of < 0.05 in univariate time-dependent analysis were included in the Cox multivariate time-dependent regression model. Multivariate Cox time-dependent regression models were calculated using hazard ratios (*HR*) and 95% confidence intervals (95% *CI*) for incident lean NAFLD (19). The Schoenfeld scale residuals test is an accurate tool for confirming proportional hazards (PH).

Third, TyG index trajectory patterns from 2017 to 2019 in the research population were characterized using semiparametric Latent Class Growth Modeling (LCGM). Briefly, the “LCTM Tools” process fitted a semiparametric mixed model using the maximum likelihood method (20). We empirically compared one, two, three, four and five group solutions and then optimized the number of subgroups by Bayesian Information Criterion (BIC) values (close to zero indicating a good fit), where the shape of trajectories was determined according to the order of the polynomial (linear, quadratic, cubic, etc.). The following parameters established the appropriate trajectory number and shape (21): (1) improvement in BIC; (2) no less than 5% membership in each trajectory group; and (3) high average group posterior probabilities (> 0.7). Four, using the multivariate Cox proportional hazard model, we created lean NAFLD prediction models to explore whether temporal patterns in these characteristics over time may enhance the model’s ability to make predictions. Based on a multivariate Cox time-dependent regression analysis, the clinical predictive model contained lean NAFLD risk variables, and the merged model incorporated metabolic factor trajectories associated with new-onset lean NAFLD. In addition, a multivariate Cox time-dependent regression model was used to adjust for potential risk factors for lean NAFLD as follows: Model 1 was univariate; Model 2 was adjusted for demographics (i.e., age, WC, and BMI); and Model 3 was adjusted for age, WC, BMI, SBP, and ALT. A spline regression model with three knots (10th, 50th, and 90th percentile) was employed to provide more accurate estimates and investigate the nature of the relationship between the TyG index and lean NAFLD.

Finally, we employed LightGBM algorithm for classification to predict the risk of lean NAFLD patients. LightGBM is a gradient-boosting framework using a tree-based learning algorithm that has been successfully applied to the construction of medical models in recent years (22, 23). Patients at the Health Management Center were randomly divided into a training set and an internal test set using R with a ratio of 7:3. To improve the effectiveness of the model while ensuring the authenticity of the data, we use a synthetic minority oversampling technique (SMOTE) for the database to solve the problem of data imbalance (24, 25). The training set was used to build the model, and the internal test set was used for model validation and evaluation. In the training set, k-fold cross-validation (k =5) is performed, and a grid search is used to find the best combination of parameters. Subsequently, the model performance is initially evaluated in the internal test set. Model performance is assessed by area under the curve (AUC), predictive accuracy, sensitivity, specificity, and F1-score. Our primary evaluation metric for ML models is AUC, calculated from ROC curves, which are graphical plots showing the diagnostic power of binary classifiers as their discriminative thresholds change (26). It is also combined with other metrics for a comprehensive assessment to determine the best model. Meanwhile, a web-based calculator was able to estimate the probability of lean NAFLD in individuals.

For all analyses, a two-tailed *P* value of < 0.05 is considered statistically significant. A general descriptive analysis was conducted on SPSS software (version 26.0; IBM, Chicago, USA). R statistical software (version 4.0.2; R Foundation for Statistical Computing, Vienna, Austria) was used to construct univariate and multivariate Cox time-dependent regression models using LCTM tools. In this study, a newly developed gradient boosting model (GBM), known as LightGBM, was implemented using “LightGBM,” packages, and “pROC,” packages to generate ROC curves, respectively. The authors make all the R and code for these tools available at www.github.com/hlennon/LCTMtools.

## Results

### The trajectory of TyG index

**Supplementary Table 1** displays the LCGM fitting results. We modeled linear, quadratic, and cubic curves from 1 to 5 classes. This table excludes five classes due to low group membership (<1%). Due to the small size of group 4 (0.27%), the quadratic curve model of group 5 was ruled out. Based on the above criteria, a cubic parametric model with 3 groups with a low BIC, relatively high posterior probability, and a high percentage of group membership was selected. The LCTM model was used to fit TyG index change trajectories to normal BMI. When divided into 4 groups, the results of the LMR-LRT likelihood ratio test had no statistical significance (*P*=0.389), and there was one group whose sample size was only 38 (< 5% of the total sample size), so it was rejected. The results showed that the optimal fitting results were divided into three groups (BIC:*P<*0.001; Entrop:*P<*0.001; LMR-LRT:*P<*0.001; Group: 206/541/363). Detailed information for calculating TyG index trajectories is provided in **Supplementary Table 1**.

**Figure 2:**
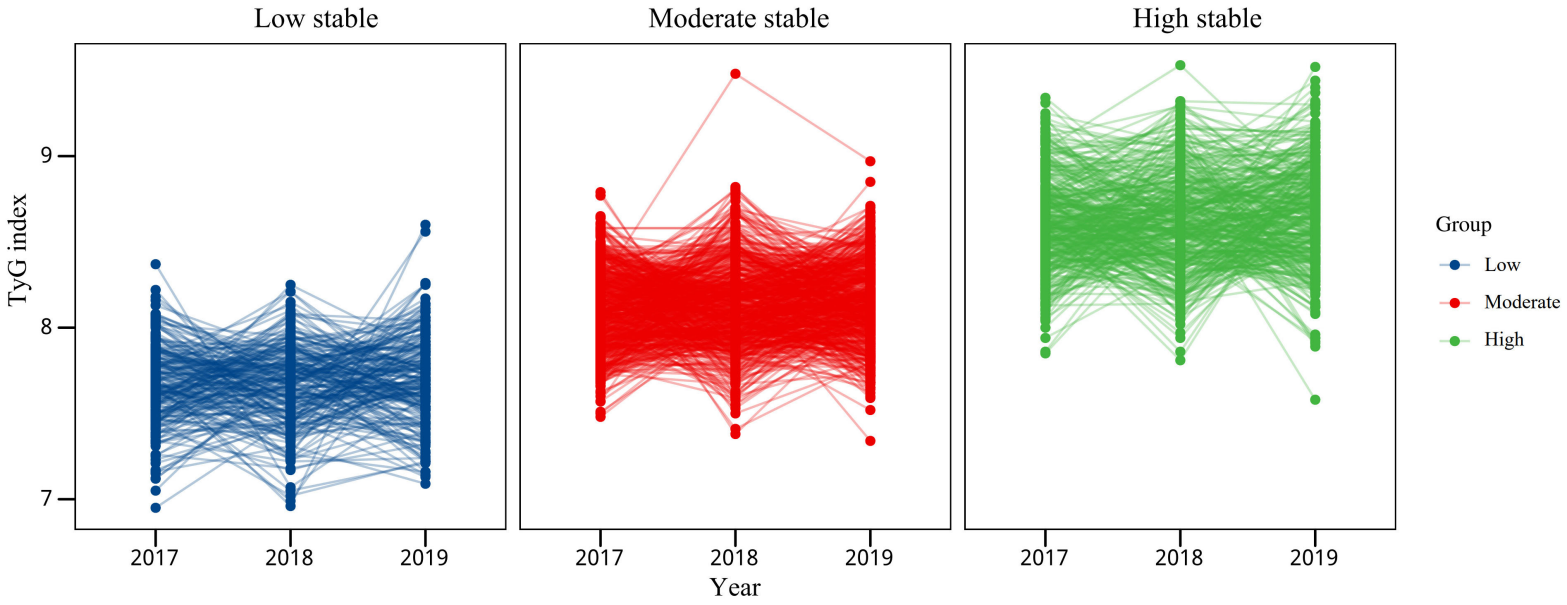
TyG index trajectories of lean NAFLD patients from the Health Management Center.

We classified the study population based on TyG index trajectories from 2017 to 2019, labeled as low stable (TyG index ranged from 7.66 to 7.71, n=206, 18.5%), moderate stable (TyG index ranged from 8.11 to 8.15, n=542, 48.8%) and high stable (TyG index ranged from 8.61 to 8.67, n=363, 32.7%). Study participants were divided into three categories based on the trajectory of their TyG index over time. According to the initial value, all change trajectories are upward trends and are divided into low stable, moderate stable and high stable (**Figure 2**).

### Characteristics of various TyG index trajectories at baseline

**Table 1** shows the demographics of the 1,109 individuals whose risk factors for developing lean NAFLD were analyzed. Over a median follow-up of 389.22 days (329.59–448.85 days), 81 (7.3%) participants developed lean NAFLD. The incidence rates of lean NAFLD were 3.8%, 8.6%, and 11.7% from low to high in men, 2.5%, 4.5%, and 11.4% in women, respectively (*P*<0.05). There were significant differences in the incidence of lean NAFLD between the three latent classes. Not only the baseline TyG index, but more importantly, TyG index trajectories in later life account for the incidence of lean NAFLD.

**Table 1:** Baseline demographic clinical characteristic according to TyG index trajectories.

Variables	Total (n=1,109)	Low-stable	Moderate-stable	High-stable	*F/χ^2^*	*P value*
male/female N (%)	441/668	53/153	209/331^a^	179/184^a^	31.36	<0.001
Age, years	46.48±14.32	40.02±12.12	46.18±14.15^a^	50.60±14.32^a,b^	38.45	<0.001
WC, cm	75.49±6.18	72.70±5.64	75.03±6.07^a^	77.76±5.82^a,b^	51.25	<0.001
SBP, mmHg	117.49±16.10	111.96±13.10	116.99±15.54^a^	121.40±17.44^a^	24.01	<0.001
DBP, mmHg	70.78±9.90	67.95±8.95	70.23±9.60^a^	73.22±10.31^a^	20.95	<0.001
BMI, kg/m^2^	21.23±1.20	20.92±1.33	21.15±1.27^a^	21.51±1.24^a^	15.82	<0.001
ALT, U/L	16.37±11.22	14.85±8.47	16.18±13.35	17.50±8.74	3.82	0.020
AST, U/L	19.28±7.01	18.76±5.70	19.23±8.46	19.65±5.03	1.08	0.340
GGT, U/L	16.67±11.41	13.66±7.19	15.52±9.78^a^	20.10±14.44^a,b^	27.49	<0.001
ALP, U/L	63.25±6.62	60.69±21.43	62.00±15.09	66.56±15.13^b^	11.36	<0.001
SUA, μmol/L	278.07±69.35	257.98±67.42	274.23±65.98^a^	295.23±71.56^a^	21.31	<0.001
TC, mmol/L	4.43(3.89,4.99)	3.95(3.60,4.48)	4.40(3.94,4.86)^a^	4.74(4.20,5.37)^a^	66.76	<0.001
TG, mmol/L	0.90(0.69,1.21)	0.57(0.50,0.66)	0.85(0.72,0.99)	1.34(1.13,1.59)	680.49	<0.001
HDL-C, mmol/L	1.51±0.33	1.64±0.34	1.56±0.33^a^	1.37±0.29	56.60	<0.001
LDL-C, mmol/L	2.69±0.70	2.21±0.51	2.62±0.62^a^	3.08±0.71^a^	126.84	<0.001
FBG, mmol/L	4.90(4.65,5.20)	4.73(4.50,4.95)	4.88(4.64,5.18)	5.05(4.77,5.34)	40.69	<0.001
HbA1c, %	5.58(5.40,5.65)	5.58(5.37,5.61)	5.58(5.40,5.64)^a^	5.60(5.40,5.83)^b^	12.75	<0.001
TyG	8.18±0.41	7.66±0.24	8.10±0.23^a^	8.61±0.26^a,b^	1022.64	<0.001

WC, waist circumference; SBP, systolic blood pressure; DBP, diastolic blood pressure; BMI, body mass index; ALT, alamine aminotransferase; AST, aspartate transaminase; GGT, γ-glutamyltransfer; HbA1c, glycated hemoglobin; ALP, alkaline phosphatase; SUA, Serum uric acid; TC, total cholesterol; TG, triglyceride; HDL, high-density lipoprotein; LDL, low-density lipoprotein; FBG, fasting blood glucose; TyG, triglyceride glucose.

The three groups were represented with a: low stable group; b: moderate stable group; If a significant level *p*<0.05 was achieved between any two of the three groups, a superscript was added to the corresponded columns.

In this study, data from the first physical exam in 2017 were used as baseline data. There were 53 males (25.7%) and 153 females (74.3%) in the low stable TyG index group, and 209 males (38.7%) and 331 females (61.3%) in the moderate stable TyG index group. There were 179 males (49.3%) and 184 females (50.7%) in the high stable TyG index group. Compared with the low stable TyG index group, age, WC, SBP, DBP, BMI, ALT, GGT, SUA, TC, TG, FPG and TyG, the stable and high stable TyG index groups significantly increased, and LDL-C significantly decreased, with statistical significance (*P*<0.001).

### Association of TyG index changing trajectory and lean NAFLD Risk

**Table 2** displays univariate time-dependent Cox regression findings. Except for sex, all variables were significantly associated with new-onset lean NAFLD (*HR*>1, *P*<0.05). **Table 3** shows the multivariate time-dependent Cox regression estimates under the PH assumption. In multivariate Cox analysis, age, WC, SBP, BMI, and ALT were correlated and independent risk factors for lean NAFLD development. Subjects in the moderate stable and high stable groups showed 1.767 (*95% CI*: 0.730-4.275) and 2.668 (*95% CI*: 1.098-6.484) fold higher risk of developing lean NAFLD, respectively, than those in the low stable group.

**Table 2:** Univariate time-dependent Cox regression analysis of new-onset lean NAFLD.

Variables	β	SE	Waldχ^2^	*HR *(95%*CI*)	*P* value
male	0.411	0.225	3.344	1.510 (0.970-2.342)	0.067
Age, years	0.031	0.007	18.604	1.031 (1.017-1.046)	<0.001
WC, cm	0.068	0.018	15.048	1.071 (1.034-1.108)	<0.001
SBP, mmHg	0.024	0.007	13.179	1.024 (1.011-1.037)	<0.001
BMI, kg/m^2^	0.350	0.085	16.734	1.419 (1.200-1.677)	<0.001
ALT, U/L	0.011	0.006	4.205	1.011 (1.001-1.023)	0.040
GGT, U/L	0.016	0.005	9.377	1.016 (1.006-1.027)	0.002
ALP, U/L	0.012	0.006	4.201	1.012 (1.001-1.024)	0.040
SUA, μmol/L	0.004	0.001	6.398	1.004 (1.001-1.007)	0.011
TC, mmol/L	0.281	0.130	4.683	1.325 (1.027-1.709)	0.030
LDL-C, mmol/L	0.353	0.147	5.720	1.423 (1.066-1.899)	0.017
FIB-4	0.171	0.150	1.300	1.186 (0.884-1.591)	0.254
FNI	2.609	3.021	0.746	13.579 (0.036-58.923)	0.388
TyG	1.078	0.256	17.727	2.938 (1.779-4.853)	<0.001

WC, waist circumference; SBP, systolic blood pressure; BMI, body mass index; ALT, alamine aminotransferase; GGT, γ-glutamyltransfer; ALP, alkaline phosphatase; SUA, Serum uric acid; TC, total cholesterol; LDL, low-density lipoprotein; FIB-4, Fibrosis-4; FNI, Fibrotic NASH Index; TyG, triglyceride glucose; NAFLD, non-alcoholic fatty liver disease; HR, hazard ratio; CI, confidence interval.

**Table 3:** Association between TyG index trajectories and lean NAFLD risk during follow-up.

Models	Total	
	*HR*(95%*CI*)	*P* value
Model 1		
low	1.000	
moderate	2.424(1.015-5.789)	0.046
high	4.524(1.921-10.655)	0.001
Model 2		
low	1.000	
moderate	1.767(0.730-4.275)	0.207
high	2.668(1.098-6.484)	0.003
Model 2		
low	1.000	
moderate	1.699(0.700-4.119)	0.241
high	2.550(1.046-6.212)	0.039

Model 1: unadjusted model.

Model 2: adjusted for age, WC, BMI, SBP.

Model 3: adjusted for age, WC, BMI, SBP, ALT.

All data are expressed as HR with 95%CI.

NAFLD, non-alcoholic fatty liver disease; HR, hazard ratio; CI, confidence interval; WC, waist circumference; BMI, body mass index; SBP, systolic blood pressure; ALT, alamine aminotransferase.

### Dose-response relationship between TyG index and lean NAFLD

**Figure 3:**
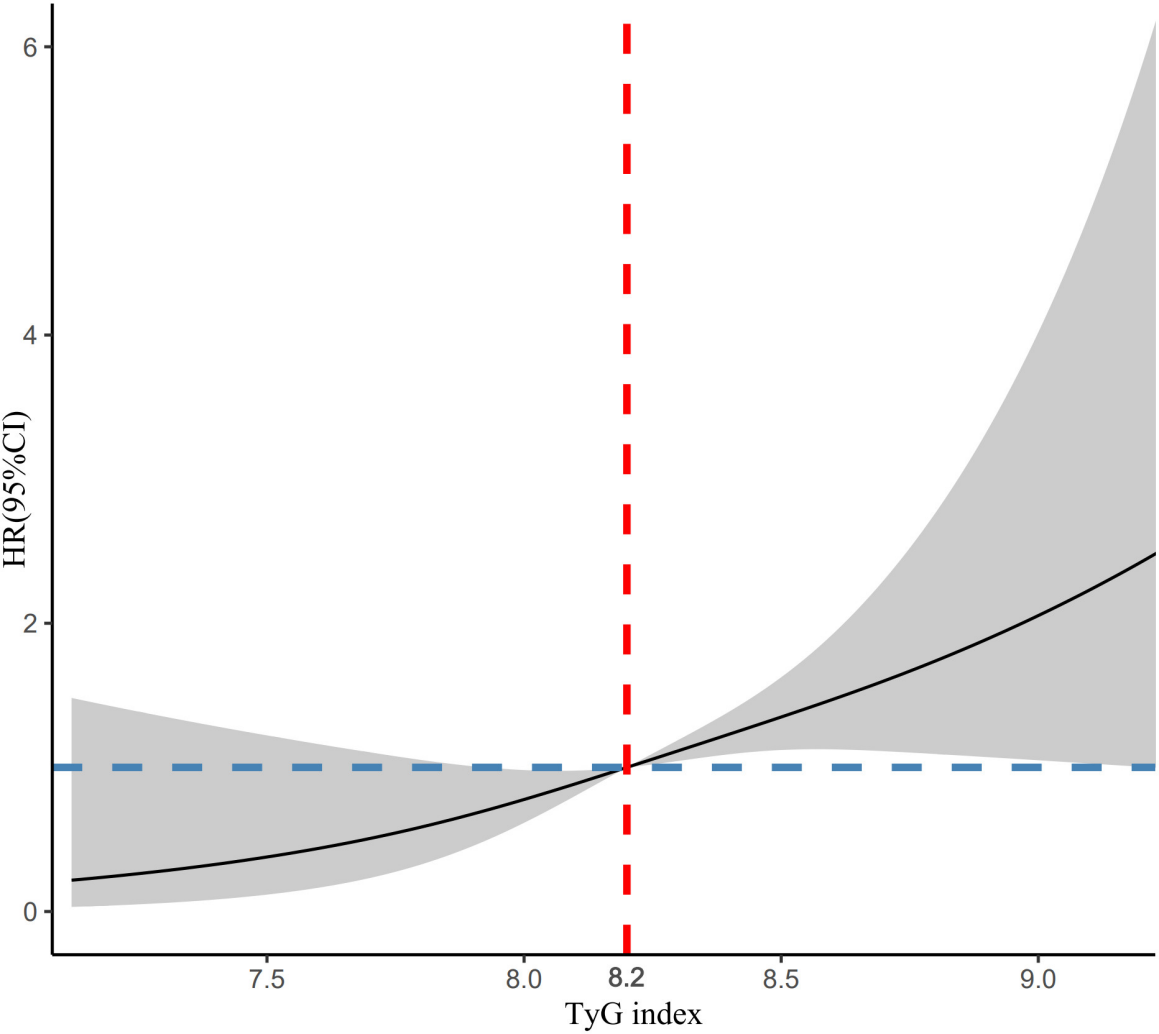
RCS plots of the association between TyG index and lean NAFLD.

The RCS model with four knots (25th, 50th, 75th, and 95th percentile) at various time periods simulated the longitudinal change in TyG index and lean NAFLD. A significant nonlinear dose-response association was found between TyG index change and lean NAFLD. After adjusting for age, WC, BMI, SBP, and ALT, these associations remained significant (*χ^2 ^= *11.5, *P*=0.003). **Figure 3** shows a positive linear dose-response relationship for TyG index and lean NAFLD. As TyG index levels increased, the detected risk of lean NAFLD showed a clear upward trend. When the TyG index exceeds 8.2, the risk of disease increases rapidly (all *p* for non-linearity > 0.1).

### The performance of machine learning model

**Figure 4:**
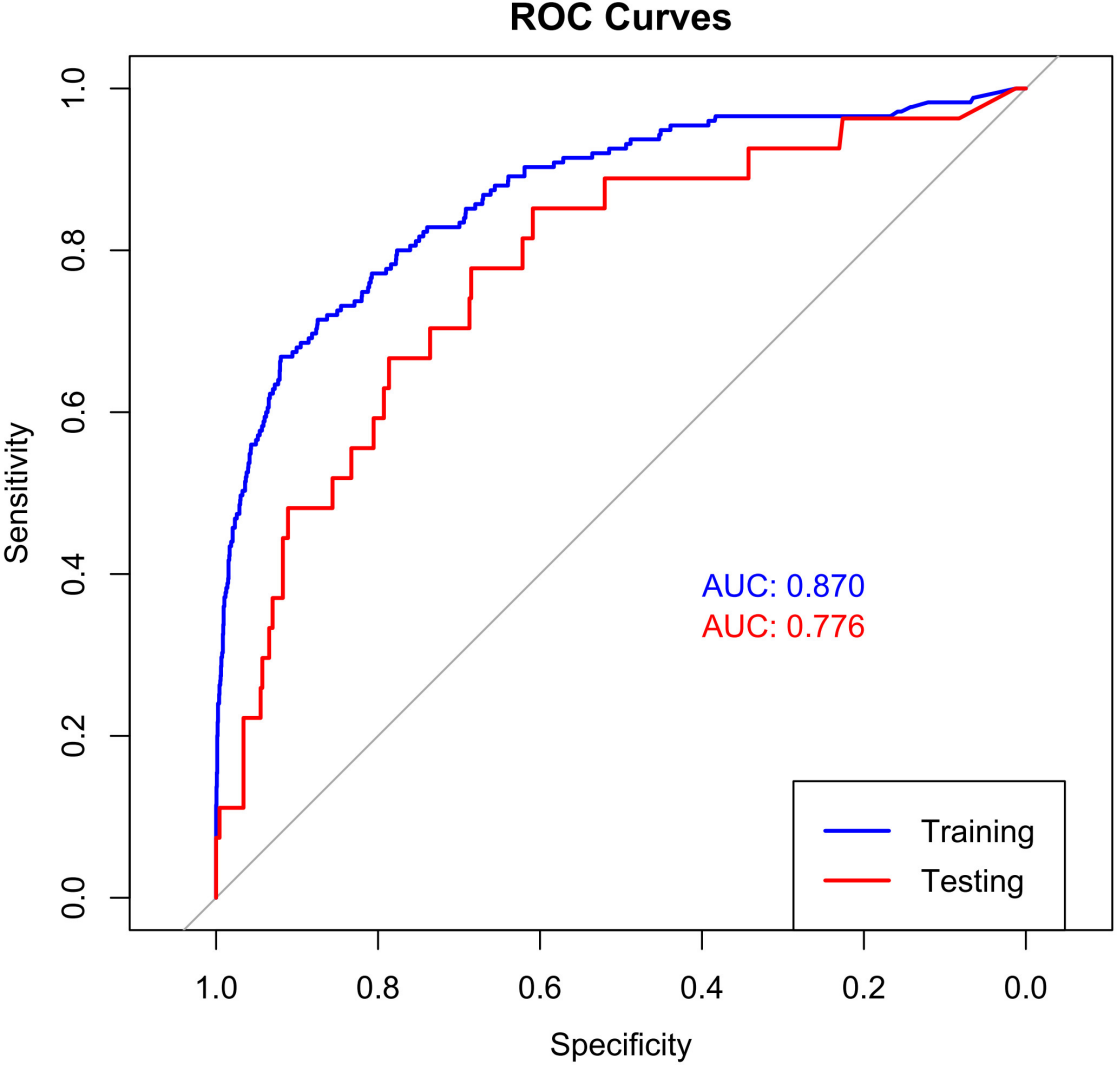
The ROC curve of LightGBM LightGBM, light gradient boosting.

The performance evaluation of the model uses the Receiver Operating Characteristic (ROC) curve and calculates the Area under the ROC (AUC) as descriptors in **Figure 4**. A higher AUC value indicates a stronger generalization capability of the model, as seen by the ROC curve approaching the top left corner of the graph. The LightGBM performs well, with AUC values all above 70% (Train AUC 0.870, Test AUC 0.766). Based on the information from the confusion matrix showing prediction results, we calculated accuracy, precision, recall, and F1 score in **Supplementary Table 2**.

### Web-based calculator

**Figure 5:**
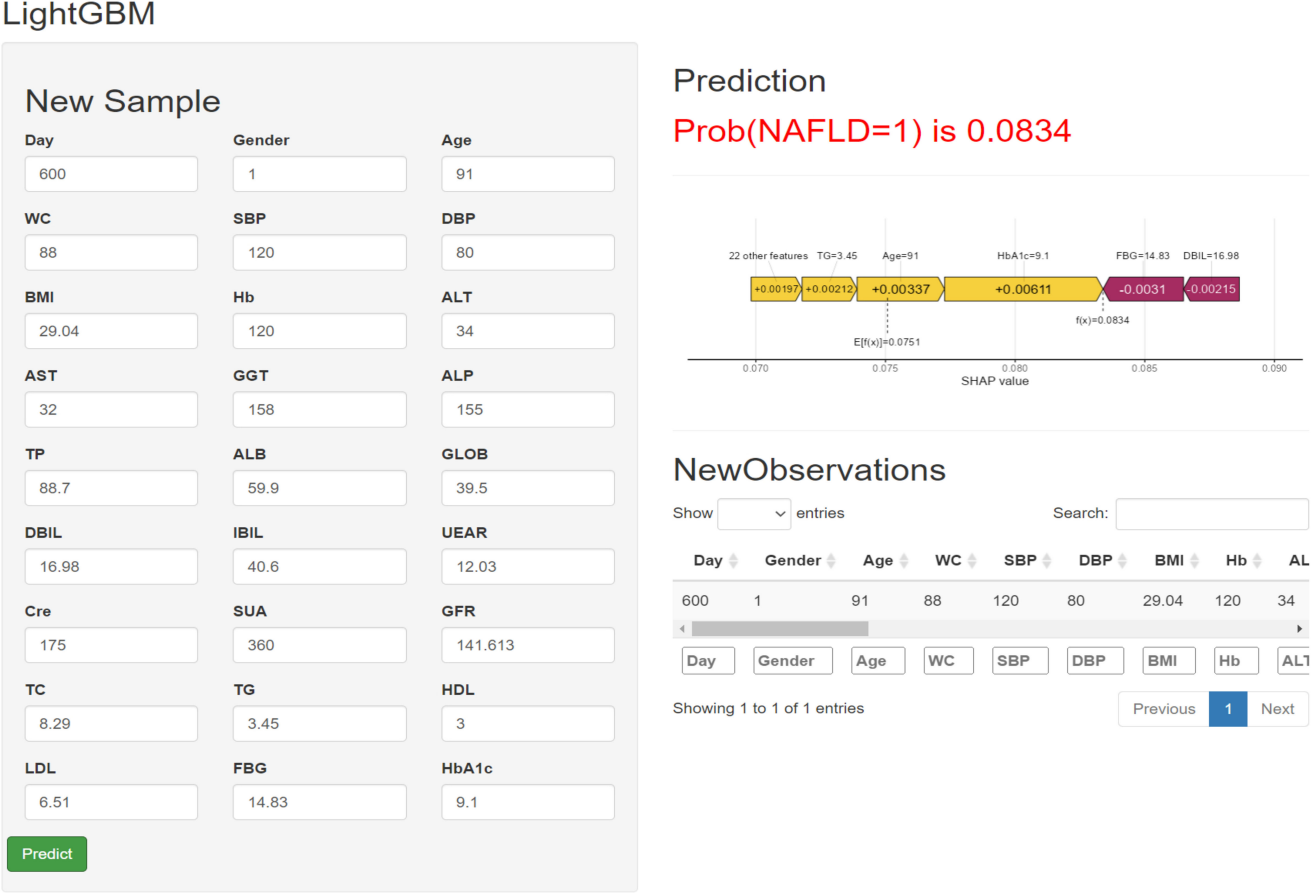
Machine learning model-based web predictor for predicting Iean NAFLD patients.

Although LightGBM has shown excellent predictive ability in lean NAFLD patients, it is intricate and complex, which is not conducive to clinical dissemination. Therefore, this study developed an online web calculator for predicting lean NAFLD patients. The calculator can be easily extended clinically and requires only the input of patient clinicopathological information to derive the probability of lean NAFLD while stratifying patients into high and low risk. (https://liuhaoshuang.shinyapps.io/lightgbm_shiny/) (**Figure 5**).

## Discussion

This study assessed longitudinal TyG index trajectories from 2017 to 2019 in a Chinese cohort using LCGM analysis. TyG index change trajectories were classified into three trajectories. In addition, higher TyG index change trajectories were positively associated with lean NAFLD risk. Age, WC, SBP, BMI, and ALT were correlated and independent risk factors for lean NAFLD in Cox multivariate studies. In routine clinical practice, the TyG index may be a valuable measure for evaluating lean NAFLD. Prior studies have established a link between TyG index and NAFLD incidence (27, 28). However, the LCGM analysis used in this study differs from previous cross-sectional and longitudinal analyses. To our knowledge, this is the first study to explore the association between TyG index trajectories and the incidence of lean NAFLD. LCGM is able to distinguish between different latent TyG indicator groups, highlighting individual differences in development. This approach differs significantly from traditional TyG index studies. In traditional studies, TyG trends in subjects are assumed to follow the same pattern, ignoring individual development. Therefore, traditional studies may not adequately reflect the association of the TyG index with incident lean NAFLD. Given these limitations, this study used machine learning techniques based on clinical indicators to develop a predictive model to identify high-risk patients. This model could help clinicians develop personalized treatment plans for lean NAFLD patients, including asymptomatic NAFLD patients. Thus, the individual TyG analysis provides a novel perspective for understanding TyG’s effect on lean NAFLD.

An important metabolic organ, the liver, controls the metabolism of lipids and glucose. The etiology and evolution of lean NAFLD are influenced by the interaction of IR and glucose/lipid dysmetabolism, which are also important variables in lean NAFLD (29). Under physiological conditions, insulin regulates glucose metabolism by processing glucose in insulin-sensitive tissues, while IR involves reduced tissue sensitivity to insulin and impaired regulation of glucose metabolism, resulting in impaired function of multiple organs, including the liver. IR causes excessive intrahepatic triglycerides by stimulating hepatic *de novo* lipogenesis (DNL) and hepatic gluconeogenesis, etc. Activated hepatic gluconeogenesis also increases glucose levels (30).

The TyG index, which is produced from fasting triglyceride and glucose levels, has been widely used as a key marker of IR, particularly peripheral and hepatic IR. Several epidemiological studies have reported a significant association of higher TyG scores with the risk of type 2 diabetes mellitus (T2DM) (31, 32), prediabetes (33), hypertension (34), cardiovascular disease (CVD) (35), coronary artery calcification (36), and polycystic ovary syndrome (PCOS) (37), which can be explained by IR. Mechanically, IR may explain the link between the TyG index and lean NAFLD. The liver functions as an endocrine organ by secreting hepatokines, metabolites, and noncoding RNA, which influence various aspects of metabolism, including glucose and lipid metabolism and insulin action. Lean NAFLD may impair its function, resulting in IR.

Our results were in line with Xue’s findings, which demonstrated that the TyG index is a valid diagnostic for detecting NAFLD in those who are neither overweight nor obese (38). In a retrospective observational study of 24,825 healthy Japanese people, Naoya et al. found similar results. They found a significant positive link between TyG-BMI and NAFLD in non-obese subjects (39). In addition, a Chinese study with a 5-year follow-up that included 841 individuals who had ultrasound revealed favorable relationships between TyG-BMI and NAFLD in the non-obese (40).

Consistent with the conventional view, this study found that BMI was associated with an increased risk of NAFLD in patients. Abdominal obesity is a significant risk factor for NAFLD (41). Waist circumference (WC) and trunk fat have been shown to significantly predict the risk of NAFLD (42). Although BMI is one of the risk factors for NAFLD, it has been argued that BMI is limited compared to other anthropometric measures (e.g., body fat distribution) in identifying lean NAFLD individuals (43).

The presence of liver fibrosis in NAFLD patients is considered the strongest predictor of long-term outcomes (44). The NAFLD Fibrosis Score (NFS), Fibrotic NASH Index (FNI) and Fibrosis-4 (FIB-4) have been recommended as appropriate methods for the initial assessment of fibrosis in NAFLD patients (17). Both methods use a combination of variables, including age, BMI, and biochemical measures (i.e., aspartate aminotransferase (AST), alanine aminotransferase (ALT), platelets, etc.). Graupera et al. concluded that NFS and FIB-4 are not optimal for screening because they do not correlate well with liver stiffness (45). In their study, WC was found to be the ideal measure for screening for fibrosis in high-risk individuals in the general population. However, other studies have found that FNI and FIB-4 have the potential to detect advanced fibrosis and fibrosis progression in people with NAFLD (46, 47). NFS and FIB-4 appear to be more useful in the diagnosis of fibrosis in NAFLD but not for screening for fibrosis in the general population. Further studies could explore a combination of these methods, including anthropometric, body composition, and biochemical variables altogether.

Recently, a Delphi multisociety conference proposed a new nomenclature for metabolic dysfunction-associated steatotic liver disease (MASLD) (48, 49). However, the shift from NAFLD to an inclusion-based definition of MASLD has yet to be extensively studied. An analysis by the LITMUS consortium showed 98% overlap between patients with conventional NAFLD and those with the newly suggested MASLD (50). However, further research is needed to adapt this new nomenclature to a specific group with lean NAFLD or SLD without any metabolic risk factors. As for prevalence, a recent meta-analysis involving 17 studies found that MAFLD has a higher prevalence than NAFLD (33.0% vs. 29.1%), and future studies should compare MASLD prevalence with NAFLD (51).

There are several limitations to this study. First, ultrasonography is used to identify lean NAFLD, but it cannot assess the degree of steatosis. Ultrasound, on the other hand, is commonly used for population-based research with acceptable accuracy. But due to the low sensitivity of ultrasound in diagnosing mild fatty liver disease, there may be a proportion of individuals in the early stages of lean NAFLD during the 2017-2019 period. And the mild lean NAFLD may have an impact on TyG’s changing trajectories from 2017 to 2019. Therefore, we cannot rule out the possibility of reverse causality between lean NAFLD and TyG index changes. Further research, including on individuals diagnosed with liver biopsies, is needed to answer this question. Second, the data came from routine health checks. The findings of this cohort may not be generalizable to the general public. Finally, our study did not consider some potential effects of unmeasured confounders on primary outcomes, including dietary changes or comorbidities. Therefore, our results should be interpreted with caution.

Even with these problems, our findings have therapeutic implications for the prevention of lean NAFLD, specifically in terms of alerting patients to TyG index levels and their shifting trajectories, and give valuable insights into the occurrence of lean NAFLD and its association with TyG index trajectories. Moreover, LCGM does not presuppose the existence of specific morphological trajectories, while it allows for distinct latent developmental trajectories that can be learned from data (52). As a result, the focus of this LCGM investigation may be to alter TyG index trajectories to isolate different, mutually exclusive groups.

This research reveals that changes in the TyG index over time are associated with an increased risk of new-onset lean NAFLD. Overall, the TyG index may be a practical and straightforward way to predict lean NAFLD, and individuals with high TyG indices should be screened for lean NAFLD and undergo extra screening and preventative measures.

## Data availability statement

The raw data supporting the conclusions of this article will be made available by the authors, without undue reservation.

## Ethics statement

The studies involving humans were approved by the First Affiliated Hospital of Zhengzhou University. The studies were conducted in accordance with the local legislation and institutional requirements. Written informed consent for participation in this study was provided by the participants’ legal guardians/next of kin. Written informed consent was obtained from the individual(s) for the publication of any potentially identifiable images or data included in this article.

## Author contributions

HL: Writing – original draft. JC: Conceptualization, Funding acquisition, Writing – review & editing. QQ: Supervision, Writing – review & editing. SY: Data curation, Writing – review & editing. YW: Methodology, Writing – review & editing. JL: Investigation, Writing – review & editing. SD: Funding acquisition, Writing – review & editing.
