# Supplementary material for: Association between TyG index trajectory and new-onset lean NAFLD: a longitudinal study
Source: Front Endocrinol (Lausanne). 2024 Feb 27;15:1321922. doi: 10.3389/fendo.2024.1321922 (PMC10927994; doi:10.3389/fendo.2024.1321922)
Supplement: Supplementary file 1 [file Table_1.docx]

**Table S1 Model fitting parameters of Latent Class Growth Models(LCMM)**

| No. Latent class | APPA | OCC | BIC | Mismatch | % Participants per class | Mean posterior probabilities in each class | % Posterior probabilities>70% |
| --- | --- | --- | --- | --- | --- | --- | --- |
| 1 | 0.804 | 16.647 | 2985.619 | -0.018 | 206/17.94 | 0.8038/0.1636/0.0034/ 0.0292 | 76.70 |
| 2 | 0.720 | 3.753 | 2946.187 | 0.065 | 541/47.13 | 0.1115/0.7201/0.1406/0.0277 | 62.85 |
| **3** | **0.782** | **7.672** | **2959.106** | **-0.003** | **363/31.62** | **0.0005/0.1190/0.7825/0.0980** | **73.28** |
| 4 | 0.824 | 56.446 | 2974.382 | -0.043 | 38/3.31 | 0.0160/0.0121/0.1479/0.8240 | 73.68 |

The best fitting model is highlighted in bold characters. After analysis by different No. of class and different initial values, quadratic 3 class model was chosen as the best fitted.

No. Latent class, latent class number of the model; APPA, Average posterior probability; OCC, odds of correct classification ;BIC, the Bayesian information Criterion; % Participants per class, proportion of participants per class.
