# Supplementary material for: Association between TyG index trajectory and new-onset lean NAFLD: a longitudinal study
Source: Front Endocrinol (Lausanne). 2024 Feb 27;15:1321922. doi: 10.3389/fendo.2024.1321922 (PMC10927994; doi:10.3389/fendo.2024.1321922)
Supplement: Supplementary file 2 [file Table_2.docx]

| **Table S2 Performances of the clinical model in the training set and test set** | | | | | | | |
| --- | --- | --- | --- | --- | --- | --- | --- |
| Clinical model | AUC(*95%CI*) | SENS | SPEC | Precision | Accuracy | Recall | F1 |
| Training set | 0.870(0.855-0.908) | 0.871 | 0.742 | 0.771 | 0.807 | 0.871 | 0.818 |
| Test set | 0.776(0.640-0.850) | 0.600 | 0.700 | 0.632 | 0.667 | 0.600 | 0.632 |

AUC, area under curve; SENS, sensitivity; SPEC, specificity; CI, confidence interval;

F1, F1 Score
